# The Effect of Bite Raise on AHI Values in Adult Patients Affected by OSA: A Systematic Review with Meta-Regression

**DOI:** 10.3390/jcm12113619

**Published:** 2023-05-23

**Authors:** Maria Lavinia Bartolucci, Serena Incerti Parenti, Francesco Bortolotti, Giulia Corazza, Livia Solidoro, Corrado Paganelli, Giulio Alessandri-Bonetti

**Affiliations:** 1 Section of Orthodontics and Dental Sleep Medicine, Department of Biomedical and Neuromotor Sciences, University of Bologna, via san Vitale 59, 40125 Bologna, Italy; serena_incerti@yahoo.it (S.I.P.); francesco.bortolott4@unibo.it (F.B.); giulia.corazza@studio.unibo.it (G.C.); livia.solidoro@gmail.com (L.S.); giulio.alessandri@unibo.it (G.A.-B.); 2 Department of Medical and Surgical Specialties Radiological Sciences and Public Health, Dental School, University of Brescia, 25121 Brescia, Italy; corrado.paganelli@unibs.it

**Keywords:** obstructive sleep apnea, mandibular advancement device, oral appliance, systematic review, meta-regression

## Abstract

Obstructive sleep apnea (OSA) is a highly prevalent sleep breathing disorder characterized by the collapse of the pharyngeal walls that entails recurrent episodes of cessation of breathing or decrease in airflow while sleeping. This results in sleep fragmentation, decreased oxygen saturation and an increase in the partial pressure of carbon dioxide, causing excessive daytime sleepiness, hypertension and increased prevalence of cardiovascular morbidity and mortality. Mandibular advancement devices (MAD) represent a valid alternative therapy to Continuous Positive Airway Pressure, thrusting the mandible forward, increasing the lateral diameter of the pharynx and reducing the collapsibility of the airway. Several investigations have focused on the detection of the best mandibular advancement amount in terms of effectiveness and tolerance, but few and contrasting data are available on the role of occlusal bite raise in reducing the apnea/hypopnea index (AHI). The aim of this systematic review with meta-regression analysis was to investigate the effect of the bite raise of MAD on AHI values in adult patients affected by OSA. An electronic search was performed in MEDLINE, the Cochrane Database, Scopus, Web of Science and LILACS. Randomized controlled trials (RCT) investigating the effectiveness of MAD in OSA patients were included. The quality of evidence was evaluated with the Grading of Recommendations Assessment, Development and Evaluation (GRADE) and the risk of bias with the Cochrane risk-of-bias tool for randomized trials (RoB2). Six RCT were included. The success rate of each study was computed: (mean baseline AHI − mean post treatment AHI)/mean baseline AHI. The GRADE scores indicated that the quality of evidence was very low. The meta-regression analysis showed that there was no correlation between the occlusal bite raise and the AHI improvement.

## 1. Introduction

Obstructive sleep apnea (OSA) is a common sleep-related breathing disorder that affects 12.5% of men and 5.9% of women over 40 years old and is characterized by recurrent episodes of partial or complete collapse of the upper airway during sleep [1]. Repetitive obstructions may lead to fragmentation of the sleep pattern, a decrease in oxygen saturation and an increase in partial pressure of CO_2_, marked swings in intra-thoracic pressure and increased sympathetic activity [2]. Consequences of OSA are represented by excessive daytime sleepiness, loss of concentration, hypertension and, in more severe cases, stroke and heart failure, resulting in an increased prevalence of cardiovascular morbidity and mortality [3].

Mandibular advancement devices (MAD) have been reported to be an alternative treatment to Continuous Positive Airway Pressure (CPAP) in patients affected by mild to moderate OSA and in those with severe OSA who refuse CPAP treatment or surgery [2,4,5,6]. These appliances work by keeping the mandible in a protruded position during sleep, increasing the lateral diameter of the pharynx, stabilizing the hyoid bone and the soft palate, stretching the muscular bundles of the tongue and preventing the posterior rotation of the mandible, thus increasing the width of the airway and reducing its collapsibility [7,8]. However, the range of success of MAD presents high variability between individuals on the basis of peculiar pathophysiological traits presented by the single patient [9]. In fact, OSA is considered a heterogeneous disorder in terms of pathogenesis and clinical manifestations. Recently, this pathology has been related to phenotypic and endotypic traits [10]. OSA patients present some degree of anatomical compromise, that puts the upper airways at risk for collapse during sleep: the association of several phenotypic factors entail the severity of such predisposition (obesity, craniofacial morphology, etc.). In addition, four non-anatomical traits emerged as playing a crucial role in the onset of OSA viz., the pharyngeal airway collapsibility, the upper airway neuromuscular compensation, the ventilator control system or loop gain and the arousal threshold. Each trait differently contributes to the clinical manifestations of OSA, influencing the individual response to the therapies. Notably, a recent review suggested that patients with mild/moderate collapsibility and with a low loop gain could benefit from MAD therapy, underscoring that endotypic traits are just one aspect to consider before prescribing a MAD therapy [11].

**Figure 1:**
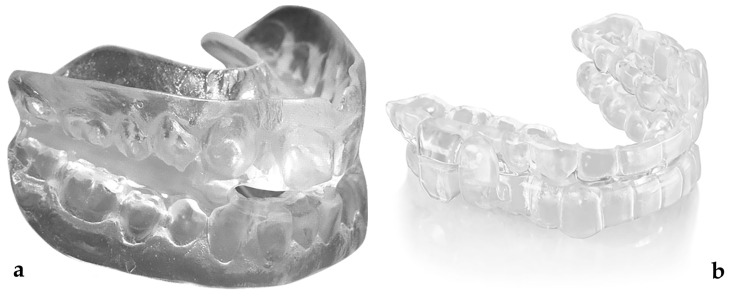
Mono-bloc MAD (**a**) and duo-bloc MAD (**b**).

Therefore, a correct diagnosis is crucial to achieving an improvement in OSA severity. Furthermore, among the selected patients, the results of the MAD therapy can be affected by the design features of these devices in terms of efficacy, tolerance and compliance [9,12,13,14,15]. A recent systematic review compared the efficacy of different types of customized MAD on reducing apnea/hypopnea index (AHI) values and improving oxygen saturation in patients affected by OSA [15]. As suggested by the authors, despite mono-bloc MAD having registered better success rate in improving the minimal oxygen saturation, the adjustability of duo-bloc MAD permits easier management of the therapy, which is reported to be better tolerated by the patients, achieving higher results in terms of compliance [15] (Figure 1).

The parameters to be followed in MAD manufacturing and the anatomical and/or dimensional changes obtained at the upper airway level are still a topic of debate. In terms of efficacy, two parameters emerged as playing a role, viz., the mandibular advancement amount and the vertical opening amount. Several studies have investigated what degree of mandibular advancement is most effective at reducing AHI value in patients affected by OSA, achieving conflicting results. Some authors suggest a mild initial protrusion [16,17], others support an initial approach with a high level of advancement [18,19,20], while stepwise mandibular advancements with progressive activations is supported by the evidence of increased compliance due to minimization of the side effects commonly brought about by MAD therapy [16,21].

A systematic review of randomized clinical trials concluded that the advancement amount does not seem to have an influence on the success rate of MAD therapy [22]. Despite this, 50% of the maximum mandibular protrusion is widely used as the minimum effective advancement to start MAD therapy, but this parameter has not been adequately addressed, since only two studies have investigated the efficacy of mandibular advancements lower than 50% [16,23]. Therefore, the most protruded mandibular position does not necessarily correspond to the best clinical outcome, and considering the amount of mandibular protrusion to be the only aspect influencing the effectiveness of MAD would be simplistic. In fact, other factors have been described as concurrently modulating the individual response to the treatment [22]. The role of the vertical increase provided by the MAD in terms of effectiveness has been little investigated. The occlusal vertical dimension is the distance measured between two points of reference when the dental arches or the upper and lower jaws are in contact. In order to determine this parameter, several methods have been proposed that can be divided as pre-extraction and post-extraction measurements. Among the pre-extraction methods, that are applicable only if the patients is dentate and with a stable occlusion, the intraoral dimensions taken clinically and on dental casts, the profile tracing, the cephalometric tracing and the phonetics are the most used. In edentulous patients, the occlusal vertical dimension can be determined by using the rest position of the subject who is asked to pronounce specific sounds, to swallow and/or to keep the mandible in a comfortable position. Other post-extraction methods provide for the evaluation of the facial esthetic appearance, of craniofacial landmarks and of phonetics. A recent review examines and discusses these different techniques, concluding that the determination of occlusal vertical dimension cannot be precise, for every available method comes along with limitations that should be taken into account by clinicians. The choice should be based on the patient’s needs and the operator’s experience [24].

A systematic review published by Ahrens et al. that investigated the efficacy of different designs of MAD emphasized the importance of considering the potential role of the amount of vertical opening in increasing the patients’ improvement [4].

Some authors have supposed that a higher vertical dimension could cause a posterior rotation of the mandibular angle and a restriction of the pharyngeal lumen [25]. Fluoroscopic recordings suggest that bite opening should be kept to a minimum because, in awake OSAS patients, it results in a posterior movement of both tongue and soft palate, with consequent narrowing of the oropharyngeal airway [26]. Similarly, Meurice et al. demonstrated in healthy subjects that mouth opening at an interincisal distance of 15 mm during sleep was associated with an increased collapsibility of the upper airway [27]. Moreover, the retracted position of the mandible caused by the rise of the vertical dimension could decrease the range of mandibular advancement; thus the efficacy of the therapy with MAD could be reduced [28]. 

However, Lowe at al. hypothesized that the mandibular protrusion, displacing the tongue away from the posterior wall of the upper airway, combined with an increased vertical dimension of occlusion, should hinder the pharyngeal closure induced by the increased vertical opening alone [29]. In addition, Pitsis et al. compared two intra-oral devices with the same degree of mandibular advancement, but with a different inter-incisal opening (4 mm vs. 14 mm); significant reductions in the AHI and in other polysomnographic variables were achieved with both oral appliances, with no statistically significant differences between the two devices, thus suggesting that the amount of the vertical opening induced by the appliances did not have a direct impact on treatment efficacy to any great extent [30].

Thus, given the wide heterogeneity of data present to date in the literature, the aim of this systematic review with meta-regression analysis is to investigate the effect of the bite raise of MAD on AHI values in adult patients affected by OSA.

## 2. Materials and Methods

The present systematic review was conducted according to the Preferred Reporting Items for Systematic Reviews and Meta-Analysis (PRISMA) system [31].

### 2.1. Search Strategy

To identify the studies to be considered for inclusion, an electronic database search of the literature was performed in MEDLINE, Cochrane Database of Systematic Reviews, Google Scholar Beta, Scopus, LILACS and Web of Sciences. Studies published before September 2022 were analyzed, and only studies written in English were included. The search strategy used for MEDLINE, including the MeSH and text words, was: ((apnea, obstructive sleep) OR (obstructive sleep apnea syndrome) OR (obstructive sleep apnea)) AND ((occlusal splint) OR (mandibular advancement device) OR (oral appliance)) AND (randomized controlled trials). Further studies were identified through hand searching the reference lists of all relevant studies.

### 2.2. Inclusion Criteria

The study selection included randomized controlled trials (RCTs) involving adult patients affected by OSA and treated with customized MAD. To be considered for evaluation, each study must report a detailed description of the appliance design, specifically the amount of occlusal bite opening, and the baseline and control PSG or sleep polygraphic parameters. Two researchers independently selected the articles. Intra-examiner conflicts were solved by discussion of each article to reach a consensus.

### 2.3. Data Items and Collection

The following data items were collected from each study: study design, sample size, mean age, mean BMI, MAD design, mean vertical opening, AHI values at baseline and after therapy, follow-up and author’s main conclusions.

### 2.4. Risk of Bias in Individual Studies and across the Studies

To document the methodological soundness of each article, the revised Cochrane risk-of-bias tool for randomized trials (RoB2) [32] was used. To evaluate the quality of body evidence, the Grading of Recommendation Assessment, Development and Evaluation (GRADE) was performed [33]. 

Two assessors independently performed the RoB2 analysis and the other two researchers performed the GRADE assessment; when in disagreement, a conjunct evaluation was performed to reach a consensus. 

The risk of bias across the studies was evaluated by means of Egger’s test and Funnel plot. Statistical tests of heterogeneity were performed to assess whether the variability in study results (effect sizes) was greater than expected to occur by chance. The heterogeneity among studies was assessed using a χ^2−^-based *Q* statistic; only an *I*^2^ index greater than 50 percent was considered and associated with a substantial heterogeneity among the studies. Moreover, the tau^2^ was calculated for the heterogeneity assessment.

### 2.5. Methodology of Synthesis of the Results of the Individual Studies

A fixed effect model was used if homogeneity across studies was proved (*p* value greater than 0.10); if homogeneity was rejected (*p*-value less than 0.10), a random effects model was used to better aggregate the data [34]. The success rate and 95 percent confidence intervals between baseline and follow-up were computed for each treatment group within the studies. A meta-analysis was conducted to analyze the effect of occlusal bite rise on success rate; the analysis was performed using Comprehensive Meta-Analysis Software v. 2.2.064 (Biostat Inc., Englewood, NJ, USA).

## 3. Results

**Figure 2:**
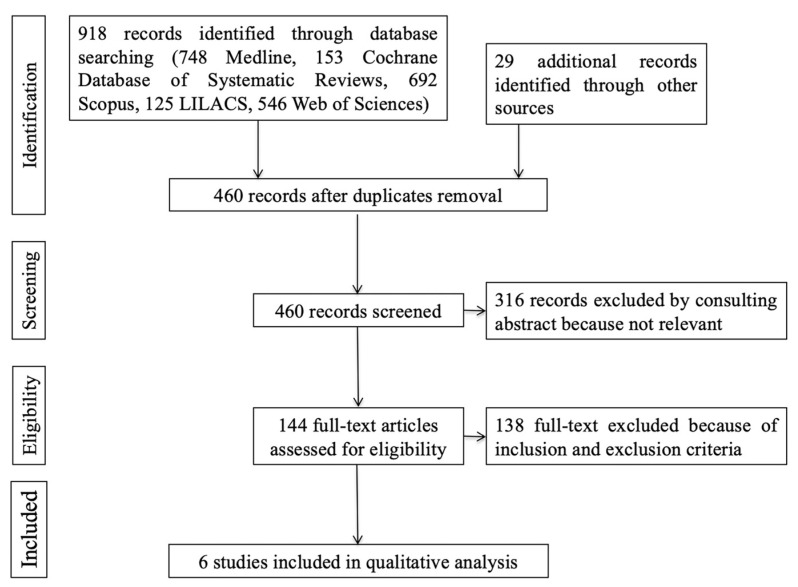
PRISMA flow diagram of the studies included in the systematic review.

Figure 2 presents the PRISMA flow diagram describing the selection process. Six studies fulfilled the inclusion criteria and were selected for the present systematic review; their main characteristics are described in Table 1. 

Since the main purpose of this systematic review was to verify the mean percentage change in AHI values before and after the raise of occlusal bite (defined as “success rate”, or the difference between mean AHI at baseline and mean AHI after treatment/mean AHI at baseline), studies that did not explicitly report these values were excluded [16,40,41,42,43,44,45,46,47].

Nikolopoulou investigated the effect of raising the bite between two groups (splint in situ versus without splint) [39] and measured the vertical bite opening considering the thickness of the acrylic splint at the level of the first molar in millimeters. Johnston and coworkers [37] used the same method to measure the bite raise caused by the MAD. In four studies the MAD was compared with a placebo device [8,36,37,38], while Blanco evaluated a single MAD with or without mandibular advancement [35]. Treatments were performed with different types of MAD and occlusal splints. In the papers by Mehta [8], Gotsopoulos [36] and Naismith [38], the occlusal vertical opening was evaluated measuring the average thickness of the acrylic appliances in millimeters. Blanco and coworkers performed measurement in the same way using a soft elastic silicone positioner.

### 3.1. Quality Analysis and Risk of Bias in Individual Studies

Table 2 shows that the risk of bias was low in three studies [35,36,39], with some concerns in two studies [37,38] and high in one studies [8]. The main reason for lowering the study quality was the missing outcome data, that is, the high drop-out rate without any analysis that estimated the effect of the deviation to the intervention.

The GRADE score for the quality of evidence was very low, as shown in Table 3. The reasons for lowering the quality of evidence were the inconsistency, the indirectness, that is the fact that most studies did not directly evaluate the bite-raising efficacy in reducing AHI values, and the risk of bias.

### 3.2. Results of Individual Studies

Table 1 reports the main results of individual studies.

Nikolopoulou et al. assessed the influence of increasing the vertical dimension of occlusion on AHI values of patients affected by OSAS: in this study, the use of stabilization splints provoked a small but significant aggravation of AHI values [39].

Blanco et al. compared the effects on AHI values using a MAD with and without mandibular advancement, obtaining a significant decrease in the mean values in both groups, with the same follow-up period, after treatment [35].

Four studies evaluated the effects of the MAD compared to a placebo device that provided only a bite raise in the control group. In two of these studies [8,37], the control group did not register significant improvements of AHI compared to baseline values, while in the others [36,38], AHI showed a reduction, albeit lower than the one obtained with the mandibular protrusion induced by MAD.

### 3.3. Synthesis of Results

**Figure 3:**
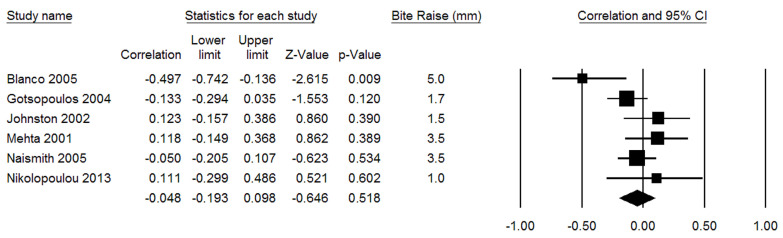
Forrest plots of success rate (correlation = success rate) [8,35,36,37,38,39].

Concerning the heterogeneity of the obtained results, a random effects model was used, resulting in *I^2^* = 50.54 and *p* = 0.072. Figure 3 shows the success rate and the confidence interval of each group included in the quantitative analysis: the overall success rate was −0.048 [−0.193–0.098] and *p* was 0.518. The effectiveness of the MAD in reducing AHI emerging from the meta-analysis of the included studies was not statistically significant. 

**Figure 4:**
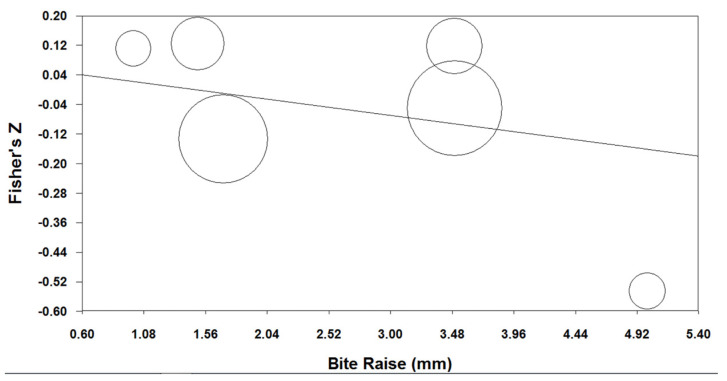
Bubble chart of the ratio between the success rate (reported as Fisher’s Z) and the amount of bite raise (size of bubbles is proportional to the weight of studies in the meta-regression).

The meta-regression analysis demonstrated the absence of a significant correlation between the occlusal bite raise (indicated in millimeters) and the success rate (Q = 1.090, *p* = 0.296), as shown in Figure 4.

### 3.4. Risk of Bias across Studies

**Figure 5:**
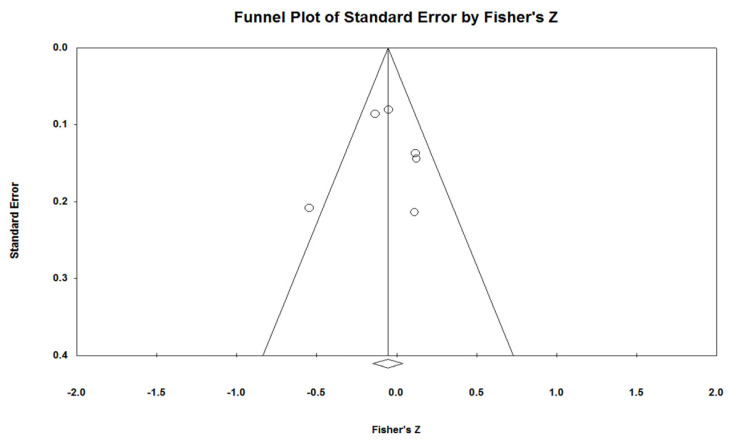
Funnel plot of the size effect of the included studies vs. standard error. Each bubble represents one study.

Concerning publication bias, the Egger’s regression test (intercept of 0.056, *p* = 0.977) demonstrated the absence of a significant deviation of the intercept from the symmetry; this means that studies with greater sample sizes were distributed close to the average, while studies with smaller sample sizes appeared to be more widely spread (Figure 5).

## 4. Discussion

The aim of the present systematic review with meta-analysis was to assess the effect of the occlusal bite raise on AHI values in adult patients affected by OSA. Only RCT were considered, and after a thorough full text analysis, only six resulted eligible for this investigation. The main outcome is represented by the absence of a significant correlation between the occlusal bite rise and the AHI improvement. 

The qualitative analysis showed that the risk of bias was low in two included studies, with some concerns in two studies and high in two studies. The main reason for lowering the quality of evidence was the missing outcome data, that is, the high drop-out rate without any analysis that estimated the effect of the deviation to the intervention.

The GRADE results indicated that the quality of the body evidence was very low for each comparison; the reasons for lowering the quality of evidence were the inconsistency, namely the heterogeneity, of the results among the studies included, and the indirectness resulting from the extent to which the studies evaluated did not directly investigate the outcome of interest of the meta-analysis, viz., the efficacy of different degrees of vertical increase in reducing AHI values.

The meta-analysis was conducted to evaluate the success rate of the therapies investigated by the selected studies, defined as the percentage of improvement of AHI post-treatment values, based on baseline values. The overall score corresponded to a reduction of 4.8%, with a confidence interval from −19.3% to +9.8% (Figure 2).

Several studies investigated the effectiveness of MAD therapy in treating OSA patients, but the literature does not provide detailed guidelines for their appropriate use, nor regarding the design of the appliance (manufacturing characteristics) that provides the best AHI improvement. Therefore, different outcomes in terms of success rate often correspond to different characteristics of the MAD used since most of the studies focused on the more effective or appropriate mandibular advancement amount to improve AHI values and clinical symptoms of patients, rarely providing explanations regarding the rationale of the protrusion rate adopted [22].

A recent systematic review evaluated the effect of MAD design on AHI reduction, sleepiness improvement, compliance, patient preferences and side effects [48]. Thirteen randomized controlled trials from 2000 to 2020 were included, and showed that MAD design features, such as the vertical dimension, need to be considered during device selection. Sari and coworkers [49] concluded that a higher vertical dimension could be associated with more TMD-related pain, while other authors indicated that a higher vertical dimension may be an important factor in increasing the airway lumen, as it causes a more pronounced stretching of the pharyngeal wall [50,51,52]. On the contrary, another study showed that a higher vertical dimension could possibly result in a reduction of the maximum protrusive position and in an increase in the posterior position or backward rotation of the mandible [28].

Pitsis et al. precisely assessed the influence of the bite opening induced by a MAD on the efficacy and side effects during OSAS treatment [30]. However, the authors did not report the posterior vertical dimension but only the interincisal one and concluded that the vertical opening had no influence on treatment efficacy [30].

The mean bite raise value of the studies evaluated in the present systematic review was 3 mm, and the meta-regression analysis showed a not-significant correlation between the bite raise (indicated in millimeters) and the success rate of MAD therapy. The regression line of the bubble chart appeared mostly flat, with no up and down trend, indicating the absence of a positive correlation between the bite raise and the success rate obtained (Figure 3). This result is in contrast with the conclusion of Gagnon et al. [53] and Nikolopoulou et al. [46], that is, occlusal stabilization splints, usually utilized for treatment of bruxism and temporo-mandibular disorders, could worsen the breathing disorders of patients affected by OSAS, since these devices could alter the upper airway patency during sleep and modify the space between dental arches.

A recent study compared three different mandibular positions using 3D volume rendering reconstructions of the pharynx in OSAS wake patients [54]. The authors noticed an increase in upper airway volume, mainly at the velopharynx region, with great variability between patients. Specifically, these study findings suggested that a minimal bite opening degree of the MAD resulted in be more effective in increasing the airway volume and inspiratory gradients compared to a larger bite-raising (15 mm) [54]. It appeared that MAD could generate a fairly significant stable pressure gradient, able to expand the pharynx volume and theoretically decrease its collapsibility. These results are in accordance with the present outcomes, as studies with low bite raise register higher improvement [8,37,39]. However, according to Op De Beeck and coworkers, in some patients, a reduction in AHI values was also achieved without evident enlargements of the pharynx, and vice versa [55]. They concluded that the patency of the airway was reduced in OSAS patients during sleep, but the treatment response to MAD was variably different among patients as the sites of collapse could be widely heterogeneous [55]. Furthermore, a recent review indicated the absence of a positive correlation between the degree of mandibular advancement and the success rate of therapy [22].

The present study was conducted with a strict methodology, and despite the analysis not showing a significant correlation among the parameters investigated, the results were equally remarkable, taking into consideration that the Egger’s test together with the funnel plot showed a low degree of publication bias, which is a form of research misconduct.

From the analysis of the available data, it emerged that only a small number of studies, with limited methodological soundness, investigated the influence of occlusal bite raise on the AHI improvement. Specifically, patients’ overbite was not related to the bite opening provided by the MAD, and no absolute values were reported, representing a limitation of the present investigation. The authors used different points of reference on the casts to measure the bite raise and also different materials in MAD manufacturing. Further improvements in the measurement of mandibular repositioning caused by the MAD are desirable with higher attention to standardize the research methodologies and to recruit homogeneous samples allowing to have comparable results and draw reliable conclusions.

Therefore, further and more methodologically sound clinical trials would be desirable to investigate the effect of occlusal bite raise on AHI values in adult patients affected by OSAS. Following the latest evidence, it emerged that many other factors could influence the individual response of patients to the treatment due to the multifactorial nature of this syndrome [9]. Therefore, the design of oral appliances should be evaluated along with other personal variables, in order to provide patients with a customized therapy, aimed at obtaining the maximum success rate with minimum side effects.

## 5. Conclusions

To date, the evidence regarding predictive factors for the individual response to MAD treatments are few and inconclusive [56]; moreover, there is low-quality evidence suggesting that the occlusal bite raise is not significantly correlated with a worsening of AHI values in OSA patients.

Thus, further well-conducted randomized clinical trials, controlling all the variables related to the design of the devices used for OSA therapy, are needed. Studies should report, in millimeters, the degree of the occlusal bite raise and the mandibular advancement, in order to overcome the results obtained from this meta-analysis and improve the evidence related to the treatment strategies for OSA.

## Figures and Tables

**Table 1:** Description of the studies included in the systematic review.

Study	Study Design	Control	Sample Size	Age (Mean ± SD)	BMI (Mean ± SD)	Degree of Vertical Opening	AHI Pre (Mean ± SD)	AHI Post (Mean ± SD)	Follow-Up	Author’s Main Conclusions
Blanco 2005 [35]	RCT	MAD with advancement vs. without advancement	7	53.0 ± 12.7	28.4 ± 4.2	5 mm	24.0 ± 12.2	11.7 ± 7.9	3 months	Mean AHI significantly decreases in both groups.
Gotsopoulos 2004 [36]	RCT cross-over	MAD vs. Placebo	67 (53 M;14 F)	48 ± 11	29.2 ± 4.8	3.5 mm	27 ± 15	24 ± 2	1 month	A reduction of 50% in mean AHI value was obtained in MAD group, while no significant reduction was seen in Placebo group (*p* = 0.18).
Johnston 2002 [37]	RCT cross-over	MAD vs. Placebo	20 (16 M;4 F)	55.10 ± 6.87	31.63 ± 5.94	1.5 mm	31.93 ± 21.18	37.68 ± 24.68	4–6 weeks	MAD is significantly more efficient in improving AHI values compared to the placebo, with a success rate of 33%.
Mehta 2001 [8]	RCT cross-over	MAD vs. Placebo	24 (19 M;5 F)	48 ± 9	29.4 ± 3.1	3.5 mm	27.0 ± 17	30.0 ± 2	5 weeks	The Placebo device has no effects on AHI values.
Naismith 2005 [38]	RCT cross-over	MAD vs. Placebo	73 (59 M;14 F)	48.4 ± 11.00	29.0 ± 5.0	3.5 mm	26.9 ± 15.4	25.4 ± 14.5	2 months	The mean AHI value is reduced of 50% in MAD group; the reduction is not significant in Placebo group (*p* = 0.18).
Nikolopoulou 2013 [39]	RCT cross-over	MAD in situ vs. non in situ	10 (3 M;7 F)	47.3 ± 11.7	<40	1 mm	15.9 ± 6.4	17.4 ± 7.0	3 nights	A small but significant increase in AHI values is noted in OSA patients with MAD in situ.

**Table 2:** Risk of bias in randomized trials (Cochrane Collaboration’s RoB 2.0 tool).

Study	Bias Arising from the Randomisation Process	Bias Due to Deviations from Intended Interventions	Bias Due to Missing Outcome Data	Bias in Measurement of the Outcome	Bias in Selection of Reported Result	Response
Blanco 2005 [35]	Some concerns	Some concerns	High risk	Low risk	Low risk	High risk
Gotsopoulos 2004 [36]	Low risk	Low risk	Low risk	Low risk	Low risk	Low risk
Johnston 2002 [37]	Some concerns	Some concerns	Low risk	Low risk	Low risk	Some concerns
Mehta 2001 [8]	Some concerns	Some concerns	High risk	Low risk	Low risk	High risk
Naismith 2005 [38]	Low risk	Some concerns	Low risk	Low risk	Low risk	Some concerns
Nikolopoulou 2013 [39]	Low risk	Low risk	Low risk	Low risk	Low risk	Low risk

**Table 3:** GRADE summary of findings for success rate.

Success Rate								
Quality Assessment						Summary of Findings		
Number of Subgroups	Risk of Bias	Inconsistency	Indirectness	Imprecision	Publication Bias (Egger’s Test)	Number of Patients	Success Rate (95% CI)	Quality
6	Serious	Serious (*I*^2^ = 50.54, *p* = 0.072)	Serious	Not serious	Not serious (*p* = 0.977)	195	−0.054 [−0.054 − 0.040]	Very Low

## Data Availability

The data presented in this study are available on request from the corresponding author.
